# “World in motion” – emulsion adjuvants rising to meet the pandemic challenges

**DOI:** 10.1038/s41541-021-00418-0

**Published:** 2021-12-21

**Authors:** Derek T. O’Hagan, Robbert van der Most, Rushit N. Lodaya, Margherita Coccia, Giuseppe Lofano

**Affiliations:** 1 GSK, Rockville, MD USA; 2 GSK, Rixensart, Belgium

**Keywords:** Adjuvants, Infectious diseases, Infectious diseases, Adjuvants, Adjuvants

## Abstract

Emulsion adjuvants such as MF59 and AS03 have been used for more than two decades as key components of licensed vaccines, with over 100 million doses administered to diverse populations in more than 30 countries. Substantial clinical experience of effectiveness and a well-established safety profile, along with the ease of manufacturing have established emulsion adjuvants as one of the leading platforms for the development of pandemic vaccines. Emulsion adjuvants allow for antigen dose sparing, more rapid immune responses, and enhanced quality and quantity of adaptive immune responses. The mechanisms of enhancement of immune responses are well defined and typically characterized by the creation of an “immunocompetent environment” at the site of injection, followed by the induction of strong and long-lasting germinal center responses in the draining lymph nodes. As a result, emulsion adjuvants induce distinct immunological responses, with a mixed Th1/Th2 T cell response, long-lived plasma cells, an expanded repertoire of memory B cells, and high titers of cross-neutralizing polyfunctional antibodies against viral variants. Because of these various properties, emulsion adjuvants were included in pandemic influenza vaccines deployed during the 2009 H1N1 influenza pandemic, are still included in seasonal influenza vaccines, and are currently at the forefront of the development of vaccines against emerging SARS-CoV-2 pandemic variants. Here, we comprehensively review emulsion adjuvants, discuss their mechanism of action, and highlight their profile as a benchmark for the development of additional vaccine adjuvants and as a valuable tool to allow further investigations of the general principles of human immunity.

## Introduction

Almost 100 years after insoluble aluminum salts (generically referred to as Alum) were established as the first adjuvants included in human vaccines, alternative adjuvants have finally received global attention and are being widely used. Since its inclusion in routine childhood vaccines, Alum has accumulated an impressive safety record, with the administration of billions of doses over many decades. However, the impressive safety record of Alum has probably served as a barrier to newer approaches. Nevertheless, Alum continues to provide an important benchmark against which all new adjuvants must be evaluated, as it is safe, effective, and well tolerated, and it remains an invaluable option^1^. The lower potency of Alum in some situations, such as in combination with vaccines containing highly purified recombinant antigens or those targeting complex pathogens, has triggered an extensive search for new and improved adjuvants. However, progress has been slow, with a single new adjuvant (MF59) included in a licensed vaccine in the late 1990s, two new adjuvants in the 2000s (AS04 and AS03), and two more since (AS01 and CpG1018)^2^. Consequently, the development of novel adjuvants has been described as one of the slowest processes in medical history^3^.

Two of the novel adjuvants currently included in licensed vaccines for use in humans are the oil-in-water emulsions MF59 and AS03. These adjuvants were widely used during the 2009 H1N1 influenza pandemic and were developed based on a long history of using emulsion adjuvants in humans, dating back to the 1930s^4^. Water-in-paraffin oil emulsions were briefly included in vaccines licensed for humans in the early 1960s but were subsequently removed from the market because of concerns around reactogenicity and safety^4^. However, the introduction of biodegradable and biocompatible oils in the 1990s once more opened the door to the use of emulsions as vaccine adjuvants^5^. The extensive experience now available with emulsion adjuvants in human vaccines attests to the efficacy and safety of this adjuvant platform, and emulsions are now being investigated in vaccines targeting SARS-CoV-2, the virus that causes COVID-19^6–10^. As a result, we have likely reached a major “tipping point”, supporting the extensive development and licensure of new vaccines containing emulsion adjuvants. In this context, it is timely to review the emulsion adjuvants currently included in licensed vaccines, i.e., MF59 and AS03 (and AF03, although this was never marketed). Here, we review how AS03 and M59 have been used to date, what we know about how they work, and the advantages that they bring.

### What is an emulsion adjuvant?

An emulsion is a mixture of two or more liquids that are normally immiscible, stabilized by the presence of a surfactant. In an oil-in-water emulsion, the oil is the dispersed phase, in water. The most commonly used oil in human adjuvants is squalene, a naturally occurring molecule found in plants and animals, including humans, in whom it is essential for the production of cholesterol, steroid hormones, and vitamin D. Squalene is a component of all the major emulsion adjuvants (Table 1)^11,12^ and is currently derived from a natural source (shark liver oil), although alternative sources are being explored^2^ and advances have been made using both synthetic biology techniques^13^ and plant sources^14,15^. Squalene-based emulsions are currently mainly manufactured using microfluidizers or high-pressure homogenization. However, emulsions can be formulated using simpler techniques such as self-emulsification^16^. It is important to highlight that squalene alone is not an adjuvant, but emulsions of squalene combined with surfactants as stabilizers can form effective adjuvants when added to antigens. Emulsion adjuvants generally do not require any physical association with the antigen, although co-administration as a mixed formulation of the two concurrently is important to performance^17,18^. AS03 contains squalene and α-tocopherol (vitamin E), an immune potentiating component, whereas MF59 comprises squalene and surfactants only^19^. Vitamins such as vitamin E have been extensively used as immune supplements^20,21^, although little is known about their mechanisms of immunomodulation. Vitamins A, B, C, D, and E, have been tested in preclinical models either as immune supplements (administered separately to a vaccine) or as adjuvants^22–27^. Vitamin E is used as an adjuvant in some veterinary vaccines^28–31^ and was initially added to AS03 due to its anti-oxidant properties, to prevent squalene degradation, but it shows immuno-potentiating effects at higher concentrations^18^. In contrast to the successful track record observed with oil-in-water emulsions, water-in-oil emulsions have also been evaluated as vaccine adjuvants but have not achieved wide success, predominantly because they are much less well tolerated.

**Table 1:** Composition of emulsion adjuvants.

Emulsion adjuvant*	Clinical development stage	Composition in a dose administered to adult humans
MF59**	Licensed for seasonal and pandemic Flu vaccine	Squalene oil (9.75 mgs); Span85 (1.18 mgs) and Polysorbate 80 (1.18 mgs) as surfactants
AS03**	Licensed for pandemic Flu vaccine, Ph III for COVID-19	Squalene oil (10.75 mgs) and α-tocopherol (11.86 mgs); Polysorbate 80 (4.83 mgs) as surfactant
Squalene-in-water emulsion (SWE)	Preclinical, Ph I for COVID-19	Squalene oil (9.75 mgs); Span85 (1.18mgs) and Polysorbate 80 (1.18mgs) as surfactants
Stable emulsion (SE) and GLA-SE or SLA-SE***	SE in Ph II for pandemic Flu; GLA-SE/SLA-SE in Ph I - Ph III for diseases such as TB, Schistosomiasis, Leishmaniasis	Squalene oil (8.6 mgs); Poloxamer188 (0.125 mgs) and synthetic phosphatidylcholines (2.73 mgs)
AF03	Was licensed for pandemic Flu	Squalene oil (12.5 mgs); Span80 (1.85 mgs) and Eumulgin B1 (2.38 mgs); also contains mannitol
CoVaccine	Ph III for COVID-19	Squalane (40 mgs); Polysorbate 80 (10 mgs) and sucrose fatty acid sulfate esters (10 mgs)

*GLA-SE* glucopyranosyl lipid adjuvant–stable emulsion, *Ph* phase, *SE* stable emulsion, *SLA-SE* synthetic lipid A stable emulsion, *SWE* squalene-in-water emulsion, *TB* tuberculosis.

*All emulsion adjuvants are either similar or lower than AS03/MF59 in droplet size.

**Both AS03 and MF59 can be and have been investigated at lower doses in clinical trials in pediatric populations^63^.

***SE composition in GLA-SE and SLA-SE is the same as stable emulsion.

### Licensed emulsion adjuvants

#### MF59

MF59 is an oil-in-water emulsion adjuvant that was first approved in Europe in 1997, in an improved influenza vaccine for adults aged 65 years and older; the adjuvanted vaccine showed enhanced functional antibody responses compared to non-adjuvanted seasonal influenza vaccines^32^. Currently licensed in more than 30 countries, the improved effectiveness of the MF59-adjuvanted versus non-adjuvanted influenza vaccine in older adults was demonstrated in a large observational study of 170,000 adults aged 65 years and over. Vaccination of older adults with MF59-adjuvanted influenza vaccine resulted in a 25% reduction in the risk of hospitalization due to influenza and pneumonia, compared with an unadjuvanted influenza vaccine^33^. In 2017, the Joint Committee on Vaccination and Immunization in the United Kingdom (UK) recommended that *Fluad* (Seqirus) should preferentially be given to subjects aged above 65, due to its established record of enhanced effectiveness^34^. In 2015, the same product was also licensed as *Fluad* in the United States (US) for use in older adults. Licensure was based on demonstrating serological non-inferiority to an already licensed influenza vaccine^35^, with evidence of superior effectiveness accumulating after licensure^36^.

This has become a common approach to licensure in the US for next-generation influenza vaccines, including those that have the potential for superiority relative to the established vaccines. The first licensure can be obtained by showing non-inferiority versus a “standard” vaccine; then, any data to show superiority, usually involving effectiveness, can be obtained during post-licensure studies. This approach has also been adopted by other manufacturers, such as Sanofi for their high-dose vaccine for older adults, with a relative efficacy trial performed post-licensure^37^, and Novavax, with their matrix-M adjuvanted vaccine^38^.

MF59 also has an acceptable safety profile in infants and young children and, compared with non-adjuvanted vaccines, significantly enhances immune responses against homologous and heterologous influenza virus strains^39^. MF59 adjuvant increased influenza vaccine efficacy from 43 to 89% in young children^40^ and is an effective adjuvant for influenza vaccines in this population^41^. Phase III studies of MF59 quadrivalent seasonal influenza vaccines in young children have been completed in Europe^42^ and are ongoing in the US (ClinicalTrials.gov Identifier: NCT01346592^43^).

MF59 also significantly improves the immunogenicity of pandemic influenza vaccines and has enabled vaccines to achieve neutralizing titers that would be expected to offer protection with lower antigen doses, and with potentially fewer doses, compared with non-adjuvanted vaccines^5^. The 2009 MF59-adjuvanted H1N1 influenza vaccine was approved in Europe for use in all persons aged 6 months and older. Approximately 100 million doses of the H1N1 pandemic vaccines containing MF59 (*Focetria* and *Celtura*, Novartis) were distributed commercially.

#### AS03

AS03 is an adjuvant system (AS) containing DL-α-tocopherol and squalene in an oil-in-water emulsion that was first developed with the aim of developing an improved influenza vaccine. However, the manufacturer, GSK, took a different approach to clinical development and committed to undertake a large phase III efficacy study in older adults. The trial was inspired by the confidence in the performance of the adjuvant based on earlier studies with avian H5N1 influenza^44^. This study (Influence65) was designed as a relative efficacy trial incorporating two consecutive influenza seasons and involving more than 40,000 adults ≥65 years of age, in stable health, located across 15 countries. Participants were randomized 1:1 to receive influenza vaccine with AS03 or the same vaccine (*Fluarix*, GSK) without adjuvant^45^. All participants were then followed through two influenza seasons (2008/2009 and 2009/2010), with a primary endpoint based on the relative rates of RT-qPCR-confirmed influenza infection (nasal and throat swabs). Unfortunately, the study failed to meet its primary endpoint, and enhanced efficacy against influenza A and/or B infection was not demonstrated. The Influence65 study illustrated the challenges associated with influenza vaccine efficacy trials, which can be confounded by multiple circulating virus subtypes, year-to-year virus strain drifts, the unpredictable effects of previous vaccination and natural exposure, along with the inability to accurately predict transmission intensity. The biggest challenge for the Influence65 study was that it began just before an influenza pandemic, which meant that the data collected for one of the years was not evaluable for the main analysis. However, even though the primary endpoint was missed, clear benefits were observed in secondary and exploratory readouts. Specifically, efficacy against A/H3N2 influenza infection as well as all-cause mortality and pneumonia was demonstrated in a post hoc analysis^45,46^. Hence, although the Influence65 study did not support licensure, the study did highlight the potential benefits of an adjuvanted influenza vaccine for older adults, even during seasons with partially mismatched influenza strains. AS03-adjuvanted influenza vaccine subsequently showed enhanced relative efficacy against H1N1 pandemic influenza in young children compared with the non-adjuvanted vaccine^47^.

The safety profile of the AS03-adjuvanted H1N1 pandemic vaccine produced in Europe (*Pandemrix*, GSK) was investigated following reports of narcolepsy in adolescents and children, primarily in Finland and Sweden^48^. Epidemiological studies revealed an increased risk of narcolepsy following vaccination with *Pandemrix*, with relative risk estimates ranging from 1.5 to 25.0 (95% confidence interval [CI] 0.3–48.5) in children and from 1.1 to 18.8 (95% CI 0.6–207.4) in adults^49^. In 2013, an increased risk of narcolepsy was also reported in the absence of pandemic vaccination, e.g., in China, Taiwan, and several European countries^50–52^, suggesting a role for the circulating H1N1 influenza virus. One hypothesis for the apparent association between H1N1 and narcolepsy was that of “molecular mimicry”, whereby CD4+ T cells to influenza hemagglutinin antigen (HA) from H1N1 (from vaccine antigen or circulating virus) cross-reacted with hypocretin, a human neuropeptide hormone, triggering narcolepsy. This hypothesis is currently being evaluated using specific HLA-DQB1*06:02 tetramers, and preliminary data indicate that antigenic mimicry exists at the CD4+ T cell level^53–55^. While further evidence is needed, a CD4+ T cell-based mechanism^54,56,57^ would be consistent with the importance of the HLA-DQB1*06:02 allele and the existence of risk alleles in the T cell receptor^58^, and would support the lack of evidence for a direct role played by the AS03 adjuvant^59^. The involvement of antigen-specific immune responses was also suggested, based on findings using a transgenic mouse model in which HA was expressed in hypocretin-secreting neurons and in which adoptive transfer of HA-specific CD4+ and CD8+ T cells triggered narcolepsy-like symptoms^60^. Other studies have identified cross-reactive antibodies between influenza virus nucleoprotein and human hypocretin receptor 2^61^, although several aspects of this work remain controversial^62,63^. A final report from the European Medicines Agency (EMA) following an evaluation of all available data stated that hypotheses involving a role for the antigens were more plausible than hypotheses based on a direct role of the adjuvant^53^.

Since 2006, more than 55,000 participants have been vaccinated with at least one dose of an AS03-adjuvanted influenza vaccine in controlled clinical trials (~25,000 with pandemic and 30,000 with seasonal influenza vaccines). In addition, during the H1N1 influenza pandemic in 2009, more than 90 million doses of the AS03-adjuvanted H1N1 vaccine were administered worldwide^49^. Following authorization, extensive pharmacovigilance data was obtained and the outcomes of these data, including post-authorization safety studies, supported a favorable benefit/risk profile for these vaccines in several populations.

#### Summary of clinical exposure to AS03 and MF59

Following the initial licensure of MF59 in an improved influenza vaccine in Europe in 1997, emulsion adjuvants continued to have a relatively low profile, with only a few million doses being administered each year in older adults. This changed dramatically during the 2009–2010 H1N1 influenza pandemic, with the distribution of a combined total of more than 190 million doses of vaccines formulated with emulsion adjuvants (MF59 and AS03)^49,64^.

Evaluations of vaccine effectiveness have shown that adjuvanted influenza vaccines (including AS03 and MF59) are significantly more effective than non-adjuvanted vaccines in children^65^ and other populations. Moreover, extensive evaluations have consistently shown that MF59 and AS03 have acceptable safety profiles^49,66–68^. Across study groups comprising different populations (children, adults, and older adults), the most frequently reported adverse events following vaccination with AS03- or MF59-adjuvanted vaccines were injection-site pain, fatigue, headache, and muscle aches. Although more reactogenic than the comparable non-adjuvanted vaccines, most symptoms were generally mild to moderate in intensity and of short duration^68–70^.

AS03 and MF59 have been widely used in licensed pandemic (H1N1) and pre-pandemic (H5N1) influenza vaccines, for stockpiling and preparedness. This widespread utilization followed demonstrations of the antigen-sparing capabilities, along with the induction of broad and potent immune responses with both AS03 and MF59^32,68,71–73^. In one study, MF59 and/or AS03 were administered with stockpiled H7N9 antigens to evaluate the potential of using one or the other of these adjuvants with a range of vaccines from different suppliers^72^. The study’s findings suggested that using adjuvants from different manufacturers with stockpiled influenza antigens was well tolerated and immunogenic. The ease of manufacturing emulsion adjuvants at scale has important ramifications when planning and implementing a rapid global response to a pandemic. Given the substantial clinical and post-licensure safety database, it is our view that emulsion adjuvants should continue to be considered the preferred adjuvants for influenza pandemic responses.

#### AF03

AF03 is an alternative oil-in-water emulsion adjuvant (Table 1). AF03 was licensed for use during the H1N1 pandemic in a vaccine called *Humenza* (Sanofi) but was never commercialized. Like other emulsion adjuvants, the AF03-adjuvanted influenza vaccine demonstrated antigen-sparing and induced a stronger immune response than the non-adjuvanted vaccine^74^. More recently, AF03 has been tested in a phase I/II clinical trial for the development of a SARS-CoV-2 vaccine^75^. AF03 is manufactured using a unique approach that has been described in the literature^76^. AF03 also constitutes the delivery system for another emulsion adjuvant, AF04, which is a formulation including a synthetic molecule (E6020) that is claimed to mimic the performance of the natural product monophosphoryl lipid A (MPL). As previously observed with glucopyranosyl lipid adjuvant (GLA)-stable emulsion (SE) adjuvant, the TLR4 agonist E6020 synergizes with the emulsion to augment antibody and cellular immune responses in mice^76,77^. GLA and second-generation lipid adjuvant (SLA) are alternative synthetic MPL-like molecules that are also TLR4 agonists. GLA and SLA adjuvants are being evaluated in emulsion formulations (SE) in clinical trials of vaccines targeting tuberculosis, schistosomiasis, leishmaniasis, and leprosy; but they have also been evaluated in H5 influenza vaccines^78,79^.

### Emulsions under development

The composition of emulsions plays an important role in their performance, but an additional way to innovate is by advancing alternative approaches to manufacturing which may have advances for widespread distribution^16,80^. As described above, emulsions can also be used to deliver additional immune potentiators^16^, an approach that has been described for SE^81,82^. SE is an emulsion adjuvant originally developed by Edgar Ribi, but subsequently advanced by the Infectious Diseases Research Institute in the US. It is similar to MF59 but contains a lower squalene content, a phospholipid emulsifier, and low concentrations of α-tocopherol (0.01% v/v)^83,84^. SE was evaluated in clinical studies of pandemic H1N1 influenza but was never licensed. However, the manufacturing process for SE was successfully transferred to alternative sites to promote local vaccine production^84^, and innovations in its manufacturing have continued, with a view to the eventual global production of pandemic influenza vaccines^7,85^.

In addition to those described above, Sepivac SWE, an alternative squalene-in-water (SWE) emulsion with the same composition as MF59, has been recently developed by researchers at Seppic and the Vaccine Formulation Institute in Switzerland; it is prepared at cGMP-grade and is being made available to the entire vaccine community under open-access terms. Sepivac SWE is currently under clinical evaluation in a seasonal influenza vaccine^86^ and in a SARS-CoV-2 vaccine^7,87,88^.

*CoVaccine HT* (Soligenix Inc.) is an alternative adjuvant, which contains sucrose fatty acid esters formulated in sub-micron size squalane-in-water emulsions^89^. Squalane is completely saturated and is more resistant to oxidation than unsaturated squalene. However, little is known about the adjuvant activity or mechanism of action of *CoVaccine HT*, although it out-performed other adjuvants used for comparison, including SE/GLA-SE^90^. *CoVaccine HT* has also been evaluated in preclinical models for a SARS-CoV-2 vaccine and showed improved immune responses when compared with Alum^91^.

### The mechanism of action of emulsion adjuvants

#### The innate immune response

Oil-in-water emulsions induce rapid recruitment of monocytes, neutrophils, eosinophils, and dendritic cells (DCs) to the injection site and subsequently to the draining lymph node (dLN). Preclinical models suggest that cellular recruitment is most likely orchestrated by the induction of chemokines, which are upregulated following exposure to emulsions^18,92^. Within hours of being injected, emulsion adjuvants induce the recruitment of antigen-loaded monocytes and DCs to the dLN^18,93^, and they rapidly accumulate in the subcapsular region^94,95^. It has been proposed that the role these subcapsular sinus macrophages (SCMs) play in mediating adjuvanticity may differ according to the type of emulsion adjuvant administered. Emulsion adjuvants may promote the accumulation of unprocessed antigen trapped in immunocomplexes within SCMs, enhancing its subsequent deposition onto non-cognate B cells and then follicular DCs, as seen with MF59^94^. Overall, these data are consistent with the central role played by SCMs in promoting immunity following administration of adjuvanted vaccines^96^.

**Fig. 1:**
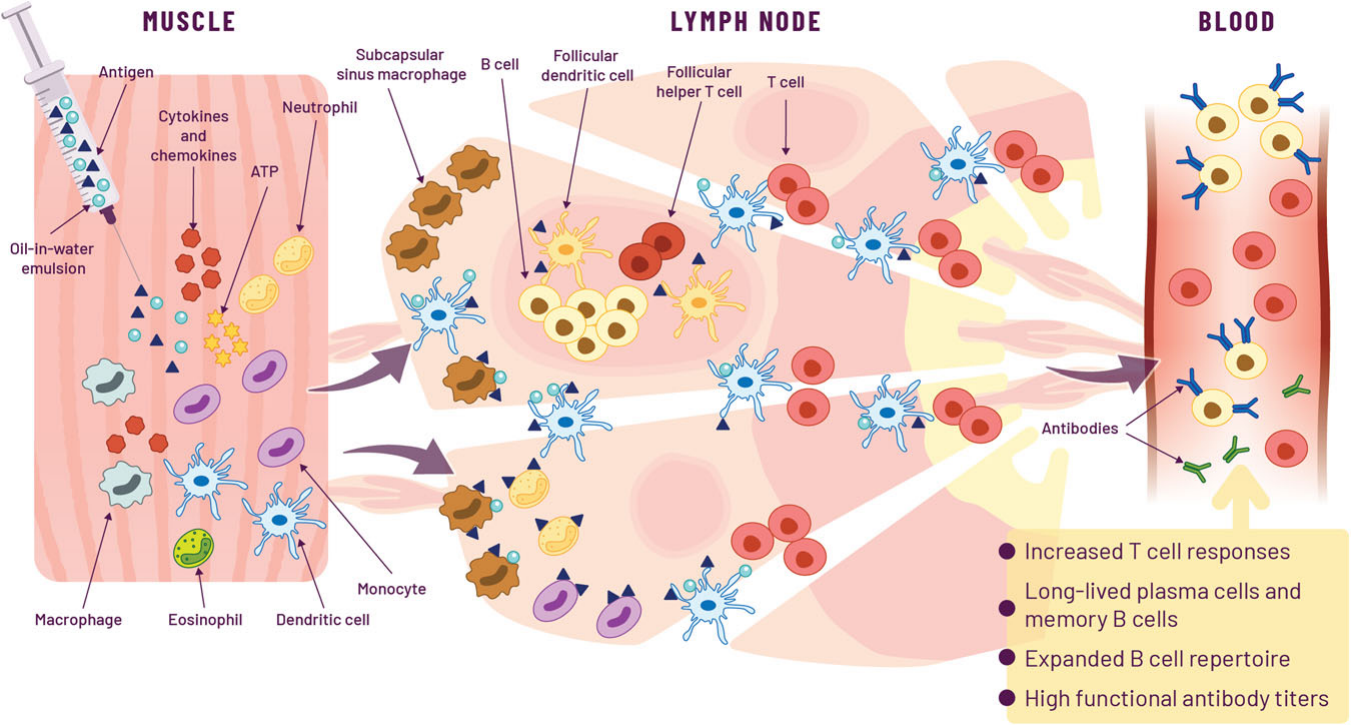
Mechanisms of action of oil-in-water emulsion-adjuvanted vaccines. Intramuscular injection of oil-in-water emulsion-adjuvanted vaccines generates an immunocompetent environment at the muscle injection site characterized by secretion of pro-inflammatory cytokines and chemokines, induction of damage-associated molecular patterns (DAMP)-signaling, and recruitment of innate immune cells such as neutrophils, eosinophils, dendritic cells, monocytes, and macrophages. Innate immune cells capture the antigen and migrate to the draining lymph node. Antigen and the emulsion adjuvant co-localize with subcapsular sinus macrophages right outside the B cell follicles, as well as with dendritic cells in the medulla. The antigen is transferred to the B cell follicles where germinal center B cells, helped by T follicular helper cells, undergo the processes of extensive somatic hypermutation and affinity maturation, and differentiate into plasmablasts or memory B cells. Antigen-loaded dendritic cells activate T cells which develop a mixed Th0/Th1/Th2 phenotype. Overall, these immunological mechanisms result in increased circulating vaccine-specific T cells, a broadly diverse repertoire of memory B cells as well as high titers of polyfunctional and cross-neutralizing serum antibodies.

In addition to mobilizing resident dLN cells, emulsion adjuvants have been shown to enhance the number of activated myeloid DCs in mouse LNs and activate human myeloid DCs in vitro^18,94^. The relative importance of the different myeloid cells in promoting immunity following immunization with emulsion adjuvants is yet to be determined. Very similar dynamics of innate immune activation in the lymph node have been described in nonhuman primates (NHPs) in comparison to mice for MF59, suggesting that the innate response to emulsion adjuvants is conserved across species^97^. Overall, the ability of emulsion adjuvants to activate innate immunity and to promote antigen uptake and presentation is likely to be central to the improved quality and quantity of the adaptive immune response observed for vaccines adjuvanted with emulsions (Fig. 1).

#### Adaptive immune response

Central to the innate–adaptive immune response axis is the enhanced induction of germinal centers (GC) and follicular helper T cells (TFHs) induced by emulsion adjuvants. MF59-adjuvanted vaccines induce high frequencies of TFHs in mice, resulting in improved formation of GCs and antigen-specific GC B cells, which can persist for at least 4 months^98^. Similar enhancement of antigen-specific B cell responses has been reported in young and aged mice^99,100^ and in infant and adult NHPs^97,101^. Similarly, AS03 promoted a strong TFH response in mice immunized with hepatitis B surface antigen (HBsAg), via an IL-6-dependent mechanism^102^, and in NHPs immunized with a nanoparticle-based SARS-CoV-2 vaccine^7^. GLA-SE was also shown to improve TFH responses^95^, but with a mechanism most likely resulting from the combined activity of the emulsion and the stimulation of TLR4^2,103,104^. TFH induction by GLA-SE was independent of SCM activation, in contrast to humoral or Th1 responses, suggesting that multiple innate immune mechanisms are required for an optimal adaptive immune response to this adjuvant^95^. Additionally, although detailed kinetics of TFH and GC induction are not available for other emulsion adjuvants, GLA-SE induced TFH and GC formation faster than other non-emulsion adjuvants, resulting in the earlier differentiation of B cells into plasma cells^105^.

In addition to inducing potent and persistent humoral responses, emulsion adjuvants also consistently induced strong CD4+ T helper cell responses in rodents^18,95,106,107^. The efficient induction of T and B cell differentiation by emulsion adjuvants results in potent antigen-specific and cross-protective humoral responses. For instance, multiple studies in ferrets have shown that AS03-adjuvanted influenza vaccines induce both heterologous and homologous antibody responses^108,109^.

#### Emulsion adjuvant-induced transcription profiles

##### MF59

Emulsion adjuvants activate multiple, distinct molecular pathways, and transcriptomics approaches have been used to shed some light on their molecular targets. The response to MF59 has been well characterized by applying microarray technology to evaluate the transcription profile at the injection site in mice. Mosca et al. demonstrated that MF59 targets muscle cells, which are activated to launch a complex pro-inflammatory response. While some of this response is shared with other adjuvants, such as CpG and Alum, the action of MF59 was more potent, resulting in the rapid recruitment of CD11b+ blood cells into muscle^92^. As the expression in muscle of genes that control leukocyte transendothelial migration was correlated with antigen-specific IgG titers in a mouse model of influenza vaccination, it is likely that MF59-mediated activation of muscle cells is important for downstream humoral responses^110^. Further studies by Vono et al. showed that MF59 induces the release of ATP from muscle cells^111^. ATP is released following cell damage and can serve as a damage-associated molecular pattern (DAMP), triggering the activation of immune cells via pattern recognition receptor (PRR) stimulation. However, the specific PRR(s) implicated in MF59 signaling have not yet been identified. MF59 does not activate Toll-like receptors (TLRs) in vitro, and MF59 adjuvanticity was preserved in knock-out mice whose macrophages lacked the ability to respond to cell damage (NLRP3−/− or caspase 1 −/− mice). However, MF59 adjuvanticity is dependent on the apoptosis-associated speck-like protein containing CARD (ASC) adaptor protein, another inflammasome component^112,113^. MF59 adjuvanticity is mediated in vivo by MyD88, an adapter protein downstream of many TLRs, although the exact function of MyD88 signaling in MF59 adjuvanticity is yet to be elucidated.

##### AS03

As with MF59, AS03 induces transcriptional changes at the injection site and the dLNs^18^. Morel et al. showed that AS03, like MF59, triggers the transient production of cytokines, leading to enhanced recruitment of granulocytes at the injection site, and dLNs, together with increased antigen-loaded monocytes in the dLNs^18^. Similar activity has been confirmed by transcriptomic analysis of peripheral blood mononuclear cells (PBMCs) in humans^114,115^. Studies with AS03 have shown that α-tocopherol is essential to enhance antigen loading in monocytes and granulocytes and support migration to the dLN^18^. As described for MF59, DAMP-mediated signaling seems to be important for AS03 adjuvanticity, specifically associated with changes in lipid cell metabolism^102^. A combination of gene expression analysis and shotgun lipidomics demonstrated that AS03 induced a rapid perturbation of lipid metabolism in monocytic cells, leading to endoplasmic reticulum stress, and activation of the unfolded protein response pathway, ultimately resulting in increased TFH responses and improved antibody avidity^102^. A distinct difference in the kinetics of the gene expression profile was recorded at the injection site and the dLN and, while a response in injected muscle was detected after 4 h, meaningful changes in the gene expression pathways linked to lipid metabolism were detected in the dLN as early as 2 h post-injection, suggesting spatiotemporal modulation of DAMP-mediated signaling following AS03 injection^102^. Lipid metabolism has also been implicated in the mechanism of action of MF59^116^; more broadly, the integration of metabolic stress signals has emerged as a critical path in the induction of enhanced responses to vaccination^117,118^ and may be generally important for lipid-rich adjuvants. Overall, although these findings do not identify direct molecular targets for emulsions, they point to an important role for DAMP-mediated signaling in the mechanisms of action for both MF59 and AS03.

##### GLA-SE

The molecular mechanism of action of GLA-SE is perhaps more clear than other emulsions, due to the inclusion of a TLR4 ligand. GLA-SE adjuvanticity is dependent on MyD88 and TRIF, both adapter molecules downstream of TLR4 activation^119^. In contrast to MF59^111^, GLA-SE adjuvanticity is dependent on interferon-alpha (IFNα) production in the dLN, presumably to trap cells by CD69 expression and to augment early innate IFNγ production by CD8+ T cells and natural killer (NK) cells. In parallel, GLA-SE induces the production of IL-12 in vivo, which synergizes with the effects of IFNα to promote the expression of the transcription factor T-bet and a Th1 commitment^120^. Transcriptomics analysis has shown that GLA-SE also induces a pro-inflammatory expression profile in the dLN, particularly centered around innate inflammation, phagocytosis, and innate immune cell migration^105^.

### Common themes and emulsion-specific immune enhancement

Common themes that emerge from the analysis of the mode of action of emulsion adjuvants are: (1) Rapid draining of the adjuvants from the muscle to the dLN, with no evidence of a “depot effect”; (2) rapid, coordinated, and potent activation of innate immune cells in the dLN; (3) increased induction of CD4+ T cells, TFHs, and GCs, resulting in a humoral immune response of enhanced quantity and quality; and (4) a complex pattern of DAMP-mediated PRR activation. Despite these common pathways, each adjuvant differs in its specific immune induction profile. As different patterns of PRR expression emerge in different innate immune cells, the activation by multiple DAMPs in emulsion adjuvants will involve different signal integration patterns, resulting in a complex inflammatory milieu.

MF59 directly activates muscle cells to induce multiple inflammatory and host defense transcriptional pathways at the injection site^92,110,111^ (Fig. 1). AS03 appears to clear more rapidly from the injection site into the dLN than MF59, a feature that has been associated with more potent induction of immune responses^121,122^. For example, AS03 was more potent in comparison with MF59 for an H7N9 influenza vaccine in man^72^. This is likely due to the additional immune potentiating effect of AS03, due to the inclusion of α-tocopherol, which was also highlighted in a study involving NHPs^7^, which compared AS03 with SWE for a SARS-CoV-2 vaccine.

Knudsen et al. compared MF59 and GLA-SE together with three other adjuvants (Alum, CAF01, and IC31), using different antigens and standardized protocols to test immunogenicity and protection in models of *Mycobacterium tuberculosis*, influenza, and chlamydia in mice^123^. Each adjuvant induced a unique “immunological signature” in each model, highlighting the importance of adjuvants in “skewing” the adaptive immune responses. MF59 and GLA-SE both induced strong antibody responses, with MF59 inducing more potent hemagglutination inhibition (HAI) titers in the influenza model. MF59 resulted in a mixed Th1/Th2 cellular response, while GLA-SE induced a more consistent Th1 bias across the different models and resulted in better protection against chlamydia, with both adjuvants reducing bacterial burden upon challenge with *M. tuberculosis*. Although designing head-to-head studies is challenging because of uncertainties regarding dose and scheduling comparisons, studies such as this are invaluable tools to help understand the potential of different adjuvants and to optimally implement the approaches available.

### Immunological features of emulsion adjuvants in humans

MF59 and AS03-adjuvanted influenza vaccines induce high titers of functional antibodies against endemic and pandemic influenza viruses in different populations, including children and older adults. Some head-to-head comparisons of MF59 and AS03-adjuvanted vaccines have been conducted, with AS03 consistently inducing the higher titers^72,124^. While HA-specific antibodies are crucial in inducing protection against influenza infection, T cells also play a critical function in promoting effector functions to limit infection, including the killing of infected target cells and orchestration of B cell responses. Both AS03 and MF59 are able to induce potent, multifunctional, and long-lasting CD4+ T cell responses following influenza immunization^69,71,125–129^. The induction of enhanced TFH responses has been demonstrated in humans for MF59-adjuvanted vaccines, and this increase was associated with improved antibody responses^128^. Similar results were obtained in NHPs for an AS03-adjuvanted SARS-CoV-2 vaccine^7,130^.

A consistent signature pattern of early post-vaccine activation of myeloid and lymphoid cells following administration of emulsion adjuvants has emerged across multiple studies in humans. Many of these data are also consistent with findings from animal models, highlighting that emulsion adjuvant-mediated immune effects are conserved across species. In both children and adults vaccinated with MF59-adjuvanted influenza vaccine, vaccination induced the expression of multiple innate immune activation transcriptional modules, suggesting the activation of monocytes, DCs, and neutrophils, as well as a strong antiviral IFN signature at day 1 post-immunization^131^. The early induction of IFN and IFN-related cytokines, such as IP-10, has also been observed with AS03 in influenza vaccine studies, and this correlated with the induction of humoral responses. Similarly, activation of myeloid and lymphoid cells 1-day post-vaccination was observed with AS03-adjuvanted vaccines^115,132–134^.

#### Cross-protection

Most influenza viruses differ in the globular head of their surface protein, HA, while the stem region is quite conserved across different strains. Therefore, humans often have preexisting immunity against HA stem epitopes. This represents a major challenge for the design of pandemic influenza vaccines because, after a lifetime of repeated exposures (through seasonal infections or vaccines), adults have an enriched pool of stem-specific memory B cells that can give rise to suboptimal responses when encountering new pandemic mutants. It is possible that a similar scenario will occur in the medium- to the long-term phase of the current SARS-CoV-2 pandemic. Indeed, given current trends, most of the global population will have developed, either through infection or vaccination, immunity against SARS-CoV-2, while still facing the threat of the emergence of new SARS-CoV-2 mutants. Emulsion adjuvants have been shown to be particularly useful in the development of vaccines against emerging viral mutants. MF59 and AS03-adjuvanted vaccines are characterized by the induction of cross-binding and cross-neutralizing antibodies with improved avidity, in addition to an expanded antibody repertoire, when compared with non-adjuvanted vaccines. Using a phage display approach to track the specificity of HA-specific antibody responses following vaccination, Khurana et al. showed that MF59 selectively enhanced antibody responses to the HA globular head, relative to the more conserved HA stem domain, as well as inducing a more diverse antibody repertoire^135,136^. Compared with the unadjuvanted vaccine, MF59 also induced expanded frequencies of memory B cells and improved affinity maturation, especially in immunologically naïve individuals, such as young children^125,136^. The characterization of serum antibody responses has confirmed the unique ability of MF59 to induce antibody diversity and cross-neutralizing responses against multiple clades of H5N1 and H1N1^137,138^. Similar immune features were observed in clinical studies with AS03-adjuvanted vaccines. In a study assessing the impact of seasonal vaccination on the immune responses to an AS03-adjuvanted H1N1 pandemic influenza vaccine, AS03 induced increased levels of vaccine-homologous and -heterologous hemagglutinin-inhibition (HI) and -neutralizing antibodies, homologous memory B cells, and plasmablasts, compared with non-adjuvanted vaccines^139^. Using the phage display approach described earlier, Khurana et al. showed that an AS03-adjuvanted H5N1 pandemic vaccine induced antibody epitope diversity to HA, with an enriched repertoire toward the HA globular head, which correlated with neutralization titers against both vaccine and heterologous H5N1 strains^140^. In a more recent study, a HA microarray was used to directly compare the cross-binding antibody responses induced by AS03 and MF59; both adjuvants induced increased broad homo- and hetero-subtypic HA responses, with AS03 achieving a higher titer and greater breadth of IgG responses relative to MF59^141^. In addition, both MF59 and AS03-adjuvanted vaccines resulted in robust HA-specific antibody-dependent cellular cytotoxicity (ADCC)-mediating antibodies against antigenically drifted H7N9 viruses^142^. A meta-analysis of available data showed that oil-in-water adjuvanted H5N1 influenza vaccines induced cross-clade reactive antibodies, although the results varied depending on the serological assay used (HAI, MN, or SRH)^143^. Given their unique ability to shape the antibody repertoire against viral mutants, emulsion adjuvants would appear to represent a key asset in the design of viral pandemic vaccines.

**Fig. 2:**
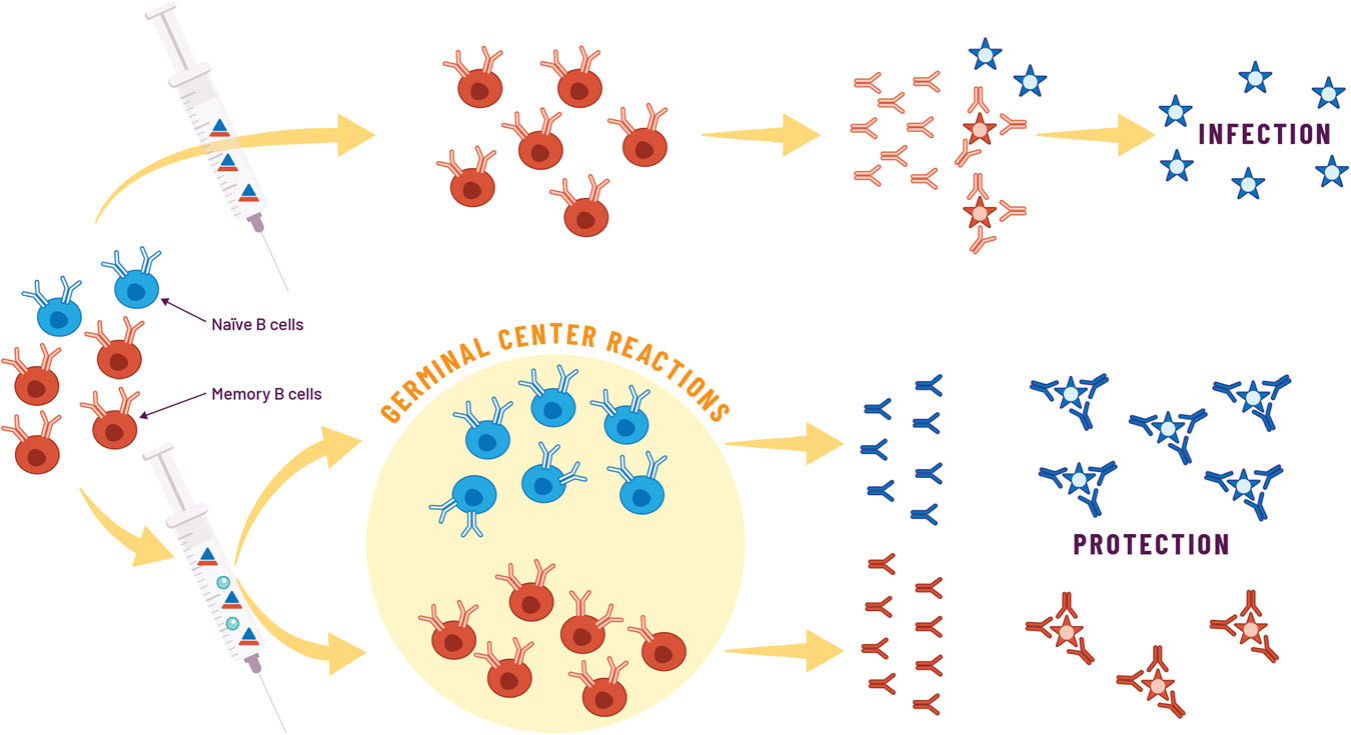
Emulsion adjuvants promote functional antibody responses against pandemic variants. An history of infection or vaccinations leads to an enrichment of circulating memory B cells that are specific toward conserved but subdominant viral epitopes. Conventional vaccination strategies aim at eliciting these memory B cells that will differentiate into plasmablasts and secrete high affinity antibodies after antigen reencounter but only against epitopes for which they had some specificity; these antibodies are typically able to prevent infection by one or few closely related viral mutants but not by very diverse mutants. By contrast, vaccination with emulsion-adjuvanted vaccines shapes the antibody responses in two ways: first preexisting memory B cells re-activate and undergo new rounds of germinal center reactions, further improving their antigenic affinity. Secondly, new naïve B cells targeting viral variants are recruited into the responses and also undergo germinal center reactions and differentiate into plasmablasts that secrete antibodies against new viral epitopes. The resulting effect is greater antibody diversity and higher overall quality of the antibody and B cell responses that are able to prevent infection by multiple and diverse viral mutants.

Several research groups have provided key insights to explain how emulsion adjuvants can achieve a broad and functional antibody diversity, revealing that these adjuvants use a bimodal mechanism. Emulsion adjuvants appear to induce the activation of naïve B cell responses against new epitopes of viral mutants combined with the re-activation of preexisting memory B cells, which further improves their affinity toward previously encountered epitopes (Fig. 2). In a study by Galson et al., the authors investigated the effect of AS03 adjuvant on the plasma cell repertoire following H1N1 influenza vaccination in individuals previously vaccinated with a seasonal influenza vaccine^144^. By analyzing mutation levels in the heavy chain variable region of the B cell receptor, they were able to distinguish sequences from cells that had been recently activated from naïve B cells, from those that were activated by memory recall, showing the combined activation of vaccine-specific naïve and memory B cells. First, AS03 stimulates increased activation of naïve B cells, thus reducing immune interference with previous vaccine responses; second, it increases the adaptability of the recalled cells to give improved specificity to the new vaccine antigen, probably through further rounds of GC reactions^145^. Ellebedy et al. reported similar findings for an AS03-adjuvanted H5N1 vaccine, providing insights into the temporal dynamics of B cell responses following a first or second vaccination^146^. The immune response to the first dose was exclusively directed at the conserved HA stem region and came from memory B cells developed through a history of seasonal vaccinations or infections; monoclonal antibodies from these memory B cells had increased levels of somatic hypermutation and recognized multiple influenza strains. The immune response following the second dose was characterized by plasmablasts with a low level of somatic hypermutation that were specific to the HA head region of the pandemic H5N1 variant, indicating that these had been recruited from a pool of naïve B cells. Importantly, the antibody response to the H5 HA stem region was much lower after the second immunization, most likely because of the blocking of these epitopes by stem-specific antibodies induced by the first immunization. Together, these data showed that emulsion adjuvants substantially increase antibody responses in humans by effectively recruiting preexisting memory B cells as well as naïve B cells to the response. Furthermore, by shaping the B cell responses toward a more diverse antibody repertoire, emulsion adjuvants may overcome the negative effect of less-functional preexisting antibodies. Consistent with the improved selection of B cells, the AS03-adjuvanted hepatitis B virus (HBV) vaccine induced superior titers of high affinity antibodies, and achieved an overall better affinity maturation than the Alum-adjuvanted vaccine in a clinical trial involving naïve adults. The same study demonstrated that AS03, similar to other adjuvant systems except for Alum, improved memory B cell quality and persistence^147^. All of these immune properties are particularly desirable in a pandemic vaccine to counter the worldwide spread of emerging SARS-CoV-2 variants.

**Fig. 3:**
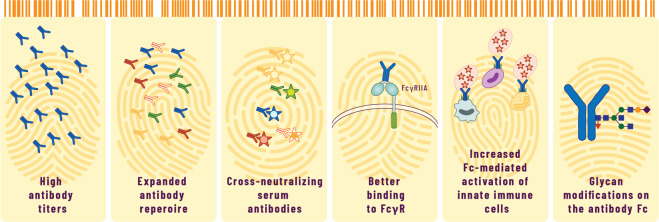
Serological barcode of oil-in-water emulsion adjuvants. The antibody responses to emulsion-adjuvanted vaccines possess peculiarities that constitute a serological fingerprint of emulsion adjuvants. As observed across multiple species and in combination with different vaccine antigens, emulsions induce high antibody titers, with a broad repertoire and a cross-neutralizing activity against multiple antigenic variants. Furthermore, systems serology analysis of serum vaccine-specific antibodies in human have revealed new important features of Fc-mediated polyfunctionality. Emulsion adjuvants modulate the Fc glycan chain of serum IgGs, induce antibodies that better bind to Fcγ receptors, and increase Fc-mediated activation of innate immune cells, including neutrophils, monocytes, and natural killer cells. Fc fragment crystallizable region, FcγR fragment crystallizable receptor for IgG, FcγRIIA Fc receptor IIA for IgG.

Systems serology analyses of the induced antibody Fc functionalities have further revealed the serological fingerprints of emulsion adjuvants, providing key insights into the mechanisms of antibody-mediated immune cell engagement and clearance of infection. Systems serology comprises a combination of high-throughput experimental techniques aimed at dissecting antibody features and functions, followed by a range of computational methods to help mine and provide an understanding of the profiled antibodies at an unprecedented depth^148^. By applying systems serology, shared serological features associated with emulsion adjuvants have been identified in human and NHP studies (Fig. 3). AS03 and MF59 induce a high degree of antibody polyfunctionality, characterized by changes in the antibody Fc glycan chain and the binding to Fc receptors, as well as in the ability of vaccine-specific antibodies to selectively recruit innate immune cells. In two separate NHP studies with MF59-adjuvanted HIV vaccines, MF59 was shown to modulate antibody Fc glycoprofiles, leading to the enrichment of sialylated vaccine-specific IgG antibodies^149,150^. Remarkably, sialylated antibodies promote enhanced immune complex deposition in GCs through complement proteins or Fc receptors, leading to the maturation of B cells with increased affinity^151,152^. These findings suggest that sialylated antibodies could play a role in mechanisms through which emulsion adjuvants modulate B cell responses and trigger repertoire diversification.

Using systems serology, Boudreau et al. assessed changes in antibody functional profiles in individuals who received H5N1 avian influenza vaccine administered with MF59, with Alum, or without adjuvant^153^. As observed in NHPs, MF59 elicited antibody responses with better binding to FcγRIIA and stimulated robust neutrophil phagocytosis and complement activity. AS03 also induced a broad spectrum of antibody functionalities, dominated by features such as the activation of phagocytosis by innate cells and of NK cell-related responses. This correlates well with the fact that IFN- and NK cell-related blood transcriptional responses were detected in the same clinical trial^115^. Notably, MF59-driven responses lacked NK cell and monocyte phagocytic features, hinting at the potential role of differential activation of Fc functions by different emulsion adjuvants. Overall, these findings point to a combination of selected serological features that represent a distinctive fingerprint of emulsion adjuvants.

### Emulsion adjuvants and the SARS-CoV-2 pandemic

The accumulated experience with emulsion adjuvants, including vaccine effectiveness, induction of cross-protective immune responses, extensive documentation of the safety profile, and ease of manufacture, including rapid scale-up, has served to highlight the potential of emulsion adjuvants for vaccine development in response to the SARS-CoV-2 pandemic. Emulsion adjuvants were previously used in the preclinical evaluation of a MERS-CoV vaccine (MF59)^154^ and a SARS-CoV-1 vaccine (AS03)^155^. In addition, *Addavax* (InvivoGen), a commercially available adjuvant that has the same composition as MF59, was used in the assessment of multiple SARS-CoV-2 vaccines in mice, guinea pigs, and ferrets^156–160^.

Efforts to develop a SARS-CoV-2 vaccine have centered around the spike protein antigen, delivered in a wild-type or stabilized form with RNA or adenovirus platforms, or as an adjuvanted recombinant protein. Both MF59 and AS03 are currently being evaluated in investigational SARS-CoV-2 pandemic vaccines. MF59 was initially tested with a recombinant SARS-CoV-2 spike glycoprotein stabilized by a “clamp” in a pre-fusion conformation. While the vaccine elicited strong immune responses and had a promising safety profile, the study was halted because the clamp antigen led to interference with diagnostic assays for HIV, a possible barrier to the widespread use of the vaccine^161^. More recently, a phase I/II clinical trial has begun to assess the safety and efficacy of IVX-411, a new, MF59-adjuvanted SARS-CoV-2 vaccine that uses a self-assembling nanoparticle with a multicopy display of the spike glycoprotein receptor-binding domain^162^. AS03 is also being tested in preclinical models of SARS-CoV-2 infection and in clinical studies. In a study by Liang et al., AS03 induced robust, high-level induction of both humoral and cell-mediated immune responses to a native-like trimeric subunit vaccine candidate for SARS-CoV-2 in rodents and NHPs^163^. In a separate study investigating the efficacy of soluble pre-fusion-stabilized spike trimer in NHPs, AS03 was critical for the induction of high-magnitude spike-specific IgG, neutralization titers, and IgA and IgG B cell responses^8^. AS03 enhanced the quality of the response, supporting the production of antibodies that mediated enhanced Fc functions. Soluble spike trimers formulated with AS03 induced a mixed CD4+ T cell response comprising Th0, Th2, and TFH. Post-challenge with SARS-CoV-2, vaccinated monkeys developed a vaccine-induced response in the lung that was able to control the infection before a significant primary T cell response could be generated. Lung mucosal IgG levels increased rapidly in vaccinated animals, as early as day 2 post-challenge, resulting in a reduction of viral replication in both the upper and lower airways. Animals receiving the highest dose of vaccine were protected from infection by day 4^8^. In a model of passive immunization, vaccine-induced IgG antibodies were sufficient to protect hamsters from the SARS-CoV-2 challenge^8^.

The value of AS03 to induce potent immune responses against SARS-CoV-2 was highlighted in a head-to-head evaluation of AS03, SWE, AS37 (TLR7 agonist absorbed to Alum), CpG1018-Alum (TLR9 agonist with Alum), and Alum adjuvants, combined with a structurally designed nanoparticle SARS-CoV-2 antigen (RBD-NP, the spike protein receptor-binding domain displayed on a two-component protein nanoparticle) in a nonhuman primate study^7^. While all five adjuvants induced neutralizing antibodies and CD4+ T cell responses following two immunizations, AS03 induced the highest neutralization titers and CD4+ T cell responses, which were once again “mixed” in profile, including mostly IL-2- and tumor necrosis factor (TNF)-α-producing cells, and TFHs. Neutralizing titers were also measured against emerging SARS-CoV-2 variants. While AS03-, AS37-, and CpG-Alum-adjuvanted vaccines all induced neutralizing antibodies against the B1.1.17 and B.1.351 variants, the AS03 group induced the highest neutralizing titer. None of the animals in the AS03 group that were challenged with SARS-CoV-2 had detectable viral RNA in pharyngeal swabs at any time, and only one animal had detectable viral RNA in nasal swabs, at very low levels. In contrast, at least one animal in all the other vaccination groups had detectable viral RNA in both pharyngeal and nasal swabs. Animals in all vaccinated groups had undetectable viral loads in their bronchoalveolar lavage. Although the number of animals per group was relatively small (*n* = 5 per group), these data suggested that uniquely among the adjuvants tested, AS03 provided protection from challenge in both the upper and lower respiratory tract. Systems serology analysis showed that all adjuvants improved the ability of antigen-specific antibodies to mediate effector functions, yet each adjuvant group mounted a distinct profile of antibody response that correlated somewhat differently with protection against the virus. For AS03 in particular, the best correlation at day 2 was found for neutralizing and binding antibodies; spike-specific IgG4, IgG2, IgG3, and IgA titers; and antibody-dependent neutrophil phagocytosis. Antibody-dependent neutrophil phagocytosis correlated with protection for SWE in this study, which had previously emerged as a key feature of the response to MF59-adjuvanted influenza vaccination^153^, raising the possibility that different emulsion adjuvants may share the ability to promote this effector function via the induction of antigen-specific antibodies. Otherwise, SWE and AS03 behaved quite differently in terms of the quality of the response induced, along with potency and efficacy, suggesting that key features of AS03 beyond the presence of squalene in the formulation mediate its functions. As discussed previously, AS03 is unique among the emulsion adjuvants in containing a significant dose of the immune potentiator α-tocopherol.

### Innate immune training to complement vaccine-specific responses

“Trained immunity” is the ability of innate immune cells to acquire memory-like capabilities following stimulation and has recently emerged as a key feature of the overall immune response against infection^164^. Trained immunity is also likely to be involved in the nonspecific protective effects of live attenuated vaccines, such as Bacillus Calmette–Guérin (BCG) and measles^165^. The capacity of adjuvanted vaccines to induce trained immunity has yet to be fully understood.

The induction of trained immunity by BCG relies on two pillars: (1) The vaccine induces strong activation of innate immunity and (2) also induces distinct epigenetic and metabolic reprogramming. Vaccination with emulsion adjuvants also results in profound changes in the innate immune cell compartment. In addition, alongside the evidence from animal models, several investigations in humans have highlighted the central role played by innate immunity in the response to emulsion-adjuvanted-vaccines, and a consistent signature of early activation of myeloid and lymphoid cells following vaccination has emerged. Additionally, an early (24 h) IFN response was observed in several studies using emulsion adjuvants and is often correlated with the later adaptive immune response^115,132–134^. Both type I and type II interferons have been shown to induce lasting and specific transcriptional memory in mouse embryonic fibroblasts, HeLa cells, and macrophages in vitro^166,167^. The totality of information known about the mechanism of action of emulsion adjuvants points to strong activation of innate immunity in humans, with some evidence supporting a role for emulsion adjuvants in modulating the epigenomic and metabolic profile of innate immune cells. Lipidomic analysis of the responses to an AS03-adjuvanted vaccine in mice hints at a role for metabolic reprogramming in its mode of action, involving pathways central to the induction of trained immunity^102,164^. Wimmers and colleagues directly assessed the effect of vaccination with the AS03-adjuvanted pandemic influenza vaccine on the epigenomic landscape of innate immune cells^168^. Similar to a trivalent inactivated vaccine, the AS03-adjuvanted H5N1 vaccine induced persistent epigenetic changes, resulting in reduced accessibility to AP-1 targeted loci and impaired cytokine response to TLR stimulation. However, in contrast to seasonal influenza vaccination, AS03-H5N1 increased the accessibility of IFN response factor loci, resulting in elevated type 1 IFN production and increased cellular resistance to viral infection in vitro. These data support the hypothesis that vaccines adjuvanted with emulsion adjuvants, and AS03-adjuvanted vaccines, in particular, can promote trained immunity. However, some crucial pieces of evidence are still missing. First, the nonspecific protective effects of adjuvanted and non-adjuvanted subunit vaccines are yet to be investigated. Post hoc analyses of the Influence65 study showed the efficacy of the AS03-adjuvanted seasonal influenza vaccine against all-cause death and pneumonia. The cause of these effects has been debated^46^, but they could at least in part be explained by the induction of trained immunity. Overall, the findings of several studies suggest that vaccines adjuvanted with emulsion adjuvants could cause a persistent change to the metabolic and epigenomic status of innate immune cells, leading to trained immunity. However, the molecular and cellular mechanisms involved remain to be elucidated and any clinical effects of such changes assessed in clinical trials.

### Emulsion adjuvants are invaluable “tools” to enable investigations into human immunity

Given the limitations of animal models in replicating human immune responses^169,170^, licensed vaccines provide a unique opportunity to better understand human immunity, since they can be used to probe the immune system of healthy individuals. Increased emphasis by regulatory authorities to understand the mechanisms of action of novel adjuvants, combined with the availability of advanced immunological readouts and -omics technologies, has enabled an unprecedented and deep scientific inquiry into the kinetics and mechanisms of the innate and adaptive immune responses that are enhanced in the presence of adjuvants. In this context, licensed adjuvanted vaccines should be considered, beyond their role in public health, as experimental tools with which to address fundamental questions about the interactions between innate and adaptive immunity. The development of noninvasive monitoring techniques, such as high-resolution positron emission tomography/computed tomography (PET/CT) scanning using tissue-specific and nonspecific radioligands and deuterium labeling of proliferating cells, allows direct in vivo or ex vivo monitoring of the nascent immune response following vaccination. For example, an exploratory PET/CT scanning study of recipients of an MF59-adjuvanted vaccine was able to recapitulate known features of the immune response to vaccination^171^. Biopsies of muscle and lymph node tissue following immunization can be done more efficiently by using radiological guidance to identify activated lymph nodes^171,172^. Non-live vaccines such as emulsion adjuvants can be used safely in immunocompromised patients and older adults^173^ and could provide a platform for exploratory trials in patients with specific deficiencies of the immune system. In these individuals, adjuvanted vaccines could be a safe proxy for infection or, more generally, immune activation, enabling the dissection of specific facets of the immune response in vivo. An-omics approach to such trials could provide insights into important immune functions, even in small trials, particularly if machine learning approaches adapted to longitudinal studies—which can reduce the impact of interindividual variability—were applied.

Vaccination of healthy individuals is also being used to interrogate relationships between immunogenicity and reactogenicity and to understand why and how adverse reactions to vaccines occur. Orthogonal experimental techniques could be combined to define individuals who experience post-vaccine reactions, which could then be validated prospectively. For instance, -omics techniques were able to identify correlates of general but not local adverse events following AS03-adjuvanted immunization^134,174^. These in vivo approaches could be complemented by ex vivo model systems to accelerate and de-risk vaccine development^175^. Additionally, more objective and precise measurements of local and systemic reactogenicity than participant-recorded diary cards could improve clinical monitoring and correlates analysis, as recently demonstrated for MF59-adjuvanted influenza vaccine^171^.

### Emulsion adjuvants are a versatile adjuvant approach for vaccine development

The accumulated evidence that we have presented here establishes that emulsion adjuvants are the best approach to take to improve influenza vaccines, including for pandemic influenza. However, there is also a considerable body of clinical evidence to suggest that emulsion adjuvants have great potential to be safe and effective adjuvants for a range of different vaccines. In an early study with a recombinant antigen (HBsAg), MF59 was shown to enable the creation of a two-dose vaccine against HBV^176^, an approach that was subsequently commercialized using an alternative adjuvant, CpG^177^. In addition, MF59 was used to show that a recombinant protein (gB) vaccine against cytomegalovirus (CMV) had significant efficacy in a phase II trial and offered protection against infection in 50% of the maternal population evaluated^178^. However, the HBV and CMV vaccines adjuvanted by MF59 were never commercialized, in common with several other vaccines that were evaluated in the clinic and showed good potency, along with an encouraging safety profile^5^. In addition, AS03 has also been evaluated in the clinic with a number of vaccines, including vaccines based on recombinant proteins and on the whole virus, and has repeatedly exhibited an excellent safety and tolerability profile, along with enhanced immune responses^49^. Hence, the use of emulsion adjuvants in licensed products^179^ and in a range of ongoing clinical evaluations^6^ highlights that they are a practical and attractive solution for the current pandemic.

### Pathways to licensure

Adjuvants contain immune stimulants and are designed to enhance the immunogenicity and efficacy of vaccines. However, theoretical concerns that the immune-stimulatory activity of adjuvants could induce or exacerbate immune-mediated diseases in susceptible individuals triggered the development of new guidelines and standardized safety monitoring by regulatory authorities and manufacturers, who were seeking to comprehensively characterize the safety profile of adjuvanted vaccines^180–182^. Regulatory guidelines issued by the World Health Organization and EMA now require that the mechanism of action of novel adjuvants on the immune response be understood as part of licensure requirements^182,183^. Authorities require the assessment of potential immune-mediated toxicity and adverse events of special interest, such as neuroinflammatory, musculoskeletal, connective tissue, and gastrointestinal disease. To this end, manufacturers have moved toward greater standardization of clinical safety monitoring, with the development of specific tools and guidelines to ensure comprehensive data capture^180,181^.

Although the current pandemic has created both the need and the opportunity for additional adjuvants to advance quickly through clinical evaluations, only Matrix-M has been so far approved for Emergency Use Authorization in Indonesia^184^ and appears close to an initial Emergency Use Authorization from the WHO. CpG1018 from Dynavax is in late-stage clinical evaluations, usually in combination with Alum, for several vaccine candidates^185,186^, while a very new adjuvant candidate, a novel TLR7/8 agonist, is being used in a product in India and appears likely to be part of the COVAX initiative for wider vaccine distribution^187^. However, based on the accumulated evidence we have summarized here, we believe that emulsion adjuvants have a much more established record of safety and high performance, due to their already extensive use in human vaccines. To summarize, this includes: (1) The inclusion of an emulsion adjuvant in a licensed product in Europe for more than 20 years; (2) the extensive use of emulsion adjuvants worldwide during a previous pandemic, i.e., the H1N1 influenza pandemic in 2009 (~200 million doses administered to diverse populations); (3) the extensive accumulated safety database over decades of use, as a consequence of (1) and (2), above; and (4) the many insights that have come from translational medicine studies in humans, which have closely interrogated the mechanism of action of emulsion adjuvants and have generally confirmed what we have learned from animal studies, while also highlighting additional opportunities to exploit the potential of emulsion adjuvants. Importantly, the manufacture of emulsion adjuvants has been shown to be robust and stable, with GSK able to commit to potentially producing 1 billion doses in 2021^188^. Moreover, it has been established through the stockpile approach of the US government as part of their influenza pandemic preparations that emulsion adjuvants can be successfully mixed with vaccine antigens from different manufacturers without impairing the performance of the vaccine candidate^124^. This capability of scale, along with a robust approach to flexible combinations, will likely be key to overcoming the challenges of the current pandemic.

## Conclusions

Emulsion adjuvants are well established and are included in licensed influenza vaccines. The safety profile of emulsion-adjuvanted vaccines has been thoroughly characterized, their potency and efficacy are better than that of non-adjuvanted vaccines, they allow dose sparing, and they can be manufactured at a large scale in response to increased demand. Emulsions are effective adjuvants in humans when combined with a range of antigens and have become the adjuvant of choice for influenza vaccines, including for pandemic responses. They consistently demonstrate the ability to induce a broad immune response against homologous and heterologous viral strains. As a consequence of these features, emulsion adjuvants are now being investigated for inclusion in protein-based vaccines as part of the medium- to long-term response to the SARS-CoV-2 pandemic. Emulsion adjuvants are capable of meeting the needs of a pandemic response that necessitates minimizing antigen content and maximizing vaccine doses (dose sparing), along with a rapid and robust manufacturing process. Emulsion adjuvants can be easily combined with recombinant proteins, engineered nanoparticles, and whole inactivated viruses, although the impact on antigen integrity needs to be monitored on a case-by-case basis. MF59- and AS03-based SARS-CoV-2 vaccines are now moving into the late stages of development^7,9,162,189,190^. Emulsion-adjuvanted SARS-CoV-2 vaccines are therefore expected to form a major part of the solution to the global pandemic.

**Fig. 4:**
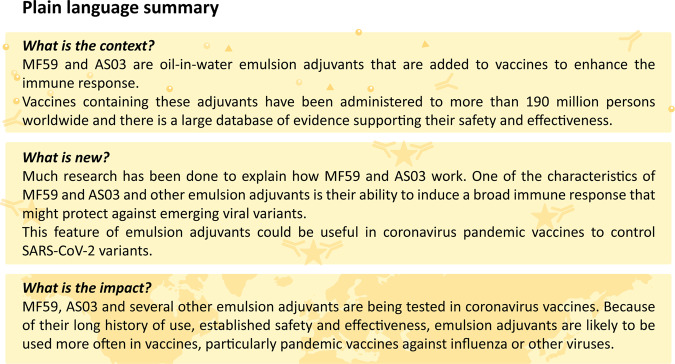
Plain language summary.

In the context of the ongoing SARS-CoV-2 pandemic, where the antigenic shift is evident, the potential for emulsion adjuvants to induce a broad, potentially cross-protective immune response could provide considerable advantages, and will continue to be closely monitored. While initial data are encouraging^191^, careful evaluation of the immune response induced by emulsion-adjuvanted vaccines relative to alternative vaccine approaches will be needed moving forward. The ability to design optimal antigenic structures through protein engineering will also be critical for success^7,192^. Two decades of global experience using emulsion adjuvants, including their key role in managing an influenza pandemic, provide a solid foundation on which to build a platform of emulsion-adjuvanted vaccines. Because of their long history of safe use in humans and their known mode of action, emulsion adjuvants represent a benchmark for the development of novel vaccine adjuvants. Emulsion adjuvants are likely to remain the preferred adjuvant for pandemic responses, including pandemics caused by influenza and other emerging pathogens. A plain-language summary of the context and main findings of this article is presented in Fig. 4.
