# Author Correction: “World in motion” – emulsion adjuvants rising to meet the pandemic challenges

**DOI:** 10.1038/s41541-023-00601-5

**Published:** 2023-02-02

**Authors:** Derek T. O’Hagan, Robbert van der Most, Rushit N. Lodaya, Margherita Coccia, Giuseppe Lofano

**Affiliations:** 1 GSK, Rockville, MD USA; 2 GSK, Rixensart, Belgium

**Keywords:** Adjuvants, Infectious diseases, Infectious diseases, Adjuvants, Adjuvants

Correction to: *npj Vaccines* 10.1038/s41541-021-00418-0, published online 21 December 2021

In the original version of this Article, a citation was misplaced that would change the meaning of the sentence. The sentence has now been updated in the HTML and PDF version of this Article with the correct citations. The conclusions of the Article are unchanged.

